# The Effects of Tai Chi Exercise Among Adults With Chronic Heart Failure: An Overview of Systematic Review and Meta-Analysis

**DOI:** 10.3389/fcvm.2021.589267

**Published:** 2021-03-18

**Authors:** Jinke Huang, Xiaohui Qin, Min Shen, Yanjuan Xu, Yong Huang

**Affiliations:** ^1^The Second Clinical Medical College of Guangzhou University of Chinese Medicine, Guangzhou, China; ^2^Department of Neurology, Guangdong Provincial Hospital of Chinese Medicine, Guangzhou, China; ^3^Department of Neurology, Zhejiang Provincial Hospital of Chinese Medicine, Hangzhou, China; ^4^School of Traditional Chinese Medicine, Southern Medical University, Guangzhou, China

**Keywords:** Tai Chi, heart failure, overview, AMSTAR-2, PRISMA, GRADE, ROBIS

## Abstract

**Background:** Tai chi (TC) is a popular form of exercise among adults with chronic heart failure (CHF), yet services are greatly underutilized. The aim of the current study was to identify and summarize the existing evidence and to systematically determine the clinical effectiveness of Tai Chi in the management of CHF using a systematic overview.

**Methods:** Both English and Chinese databases were searched for systematic reviews (SRs)/meta-analyses (MAs) on TC for CHF from their inception to June 2020. The methodological quality, reporting quality, and risk of bias of SRs/MAs were assessed using Assessing the Methodological Quality of Systematic Reviews 2 (AMSTAR-2), the Preferred Reporting Items for Systematic Reviews and Meta-Analyses (PRISMA) checklist, and Risk of Bias in Systematic reviews (ROBIS), respectively. The evidence quality of outcome measures was assessed by the Grades of Recommendations, Assessment, Development and Evaluation (GRADE).

**Results:** Six SRs/MAs using a quantitative synthesis to assess various outcomes of TC in CHF were included in this overview. The methodological quality, reporting quality and risk of bias of the SRs/MAs and the evidence quality of the outcome measures are generally unsatisfactory. The limitations of the past SRs/MAs included the lack of either the protocol or registration, the list of excluded studies, and the computational details of meta-analysis were inadequately reported. The critical problems were that qualitative data synthesis relied on trials with small sample sizes and critical low quality.

**Conclusions:** TC may be a promising complementary treatment for CHF. However, further rigorous and comprehensive SRs/MAs and RCTs are required to provide robust evidence for definitive conclusions.

## Introduction

Heart failure (HF) is a serious clinical syndrome caused by a variety of structural and functional cardiac disorders that result in the inability of the heart to meet the body's needs (1). At least 26 million people suffer from HF worldwide, and the prevalence is increasing owing to an aging population (2). Moreover, HF imposes a significant economic burden, which is estimated at $108 billion per annum (3). Due to its high morbidity and mortality, HF has become a public health problem that seriously affects patients' health (2). Dyspnea and fatigue are two of the most debilitating symptoms in patients with chronic heart failure (CHF) (4); these individuals frequently experience low exercise tolerance, poor quality of life (QoL), and recurrent hospitalizations and are at greater risk for morbidity and mortality (5, 6).

Exercise-based cardiac rehabilitation is an effective means to improve the QoL of patients with CHF with improved exercise tolerance and fewer CHF-related hospitalizations reported (6, 7). In addition, cardiac rehabilitation in CHF patients helps prevent social isolation (5). Moreover, cardiac rehabilitation (with exercise training at its core) has become an important recommendation in clinical guidelines (8). As a low-intensity, low-impact physical activity that originated from China, Tai Chi (TC) is suitable for older adults to perform, including those with poor exercise tolerance or chronic health conditions (9). It is believed that TC may be a promising adjunct to exercise-based cardiac rehabilitation in adults with CHF (10).

A literature search yielded several published systematic reviews (SRs)/meta-analyses (MAs), and the results revealed that the application of TC in the management of CHF has already been addressed. Although SRs/MAs are important tools to guide evidence-based clinical practice, their quality has been criticized in multiple medical fields (11, 12). An overview of SRs/MAs is a relatively new method to synthesize the outcomes of multiple SRs/MAs, appraise their quality and to attempt resolve any discordant outcomes (13). The aim of this study was to assess the scientific quality of past SRs/MAs regarding the application of TC in the management of SRs/MAs using a systematic overview.

## Methods

The current study adheres to the guidelines for systematic reviews according to the Cochrane Handbook (14), and Preferred Reporting Items for Systematic Reviews and Meta-Analyses (PRISMA) (15). The literature search, literature selection, data extraction, and quality evaluation were done by both two reviewers independently and any inconsistencies were resolved through consensus or by consulting an experienced third reviewer.

### Inclusion and Exclusion Criteria

The inclusion criteria were as follows: (a) study design: SRs/Mas based on random control trails (RCTs) in which the participants were patients with CHF and were diagnosed according to any internationally recognized clinical guidelines; (b) intervention: TC combined with conventional medication (CM) vs. CM alone; (c) outcomes: 6-min walk distance (6MWD), QoL (applying the Minnesota Living with Heart Failure Questionnaire, MLHF), serum B-type natriuretic peptide or N-terminal pro brain natriuretic peptide (BNP or NT pro-BNP), left ventricular ejection fraction (LVEF), peak oxygen uptake (peak VO_2_), systolic blood pressure (SBP), diastolic blood pressure (DBP), heart rate(HR). Non-RCT SRs/MAs, repeated publications, review comments, conference abstracts, editorials, and guidelines were excluded.

### Search Strategy

We searched PubMed, EMBASE, the Cochrane Database of Systematic Reviews, Web of Science, China National Knowledge Infrastructure, Sino-Med, Chongqing VIP, and Wanfang Data databases from inception to June 2020. We used the following search strategy: (heart failure OR cardiac failure OR decompensation heart OR myocardial failure) AND (Tai Chi OR Tai Ji) AND (systematic review OR meta-analysis) as subject word and random word for all fields.

### Eligibility Assessment and Data Extraction

The titles and abstracts of all articles were screened firstly, and potentially eligible articles were retrieved for perusal in full text. A standardized form was designed to extract the following information from each eligible review: first author, publication year, country, number of RCTs enrolled, quality assessment tool for RCTs enrolled, interventions in treatment and control groups, outcome measures, data synthesis methods, and main conclusions.

### Review Quality Assessment

Assessing the Methodological Quality of Systematic Reviews 2 (AMSTAR-2) (16) was used to assess the methodological quality of each SR/MA based on the following domains: (a) preparation for review, (b) search for and selection of primary studies, (c) data coding and reporting, (d) data synthesis. It consists of 16 items, and seven of them were critical domains. Each item was evaluated using three evaluation options, yes (indicating high quality), partial yes (partial quality) or no (poor quality).

Preferred Reporting Items for Systematic Reviews and Meta-Analyses (PRISMA) checklist (15) was applied to assess report quality of each SR/MA based on the following domains: (a) title, (b) abstract, (c) introduction, (d) methods, (e) results, (f) discussion, (g) funding. It consists of 27 items focusing on the reporting of methods and results in a meta-analysis.

Risk of Bias in Systematic reviews (ROBIS) (17) was used to assess the risk of bias of each SR/MA based on the following domains: (a) Phase 1 assessing relevance, (b) Phase 2 covers 4 domains through which bias may be introduced into an SR: Domain 1 “study eligibility criteria,” Domain 2 “identification and selection of studies,” Domain 3 “data collection and study appraisal” and Domain 4 “synthesis and findings,” (c) Phase 3 assesses the overall risk of bias in the interpretation of review findings and whether this considered limitations identified in any of the phase 2 domains.

The Grades of Recommendations, Assessment, Development, and Evaluation (GRADE) (18) was used to assess the evidence quality of each outcome measure enrolled in these SRs/MAs based on the following domains: (a) risk of bias (that is study limitations), (b) inconsistencies, (c) indirectness, (d) inaccuracy, (d) publication bias.

### Data Synthesis and Presentation

A narrative synthesis was used in this overview. The characteristics and results of each SR/MA as well as the results of AMSTAR 2, PRISMA and ROBIS were summarized by tabulation and figures. The GRADE evidence profile and summary of findings table were generated by using the GRADE pro GDT online software.

## Results

### Results on Literature Search and Selection

**Figure 1:**
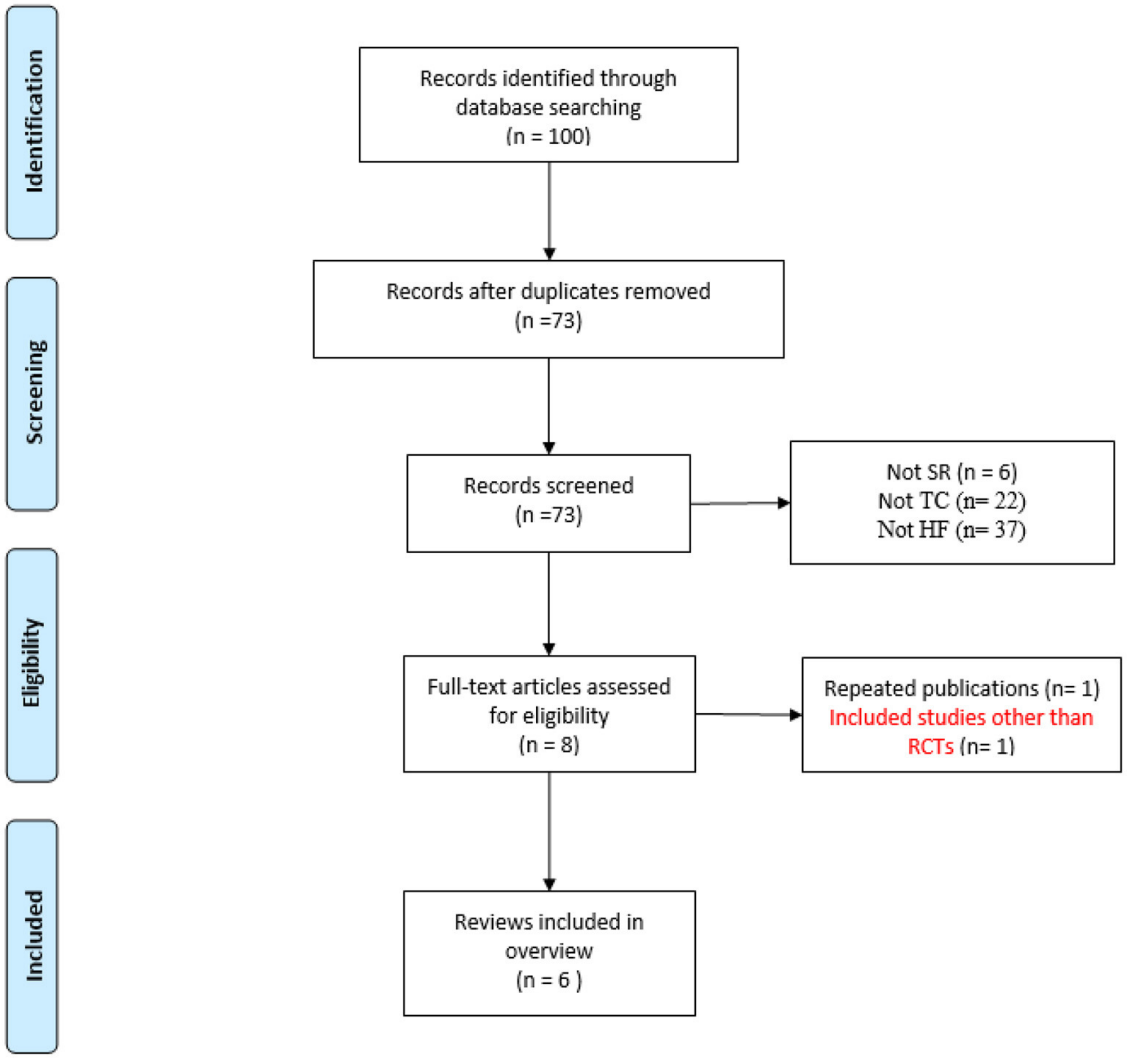
Flow diagram of the literature selection process.

A total of 100 records were identified through electronic search. After duplicates were removed, the titles and abstracts of 92 records were screened. Afterwards, 8 manuscripts were included for full-text reading, of which 2 studies were excluded because 1 record was a repeated publication and the other included studies that were not strictly RCTs. Finally, 6 SRs/MAs (19–24) were included in the current overview. The flowchart of the study selection is shown in Figure 1.

### Description of Included Reviews

The 6 included SRs/MAs were published between 2013 and 2020, including 5 articles from China and 1 from America. Four articles were published in English and the remaining 2 were in Chinese. All reviews included only RCTs and conducted a meta-analysis. The number of RCTs included in each MA ranged from 4 to 11, and individual study sample sizes ranged from 229 to 904. The quality assessment scales of the original studies varied: 1 used Downs and Black Quality Index checklist, 4 used Cochrane risk of bias criteria, 1 adopted the modified Jadad scale. The intervention measures were TC plus CM in the treatment group, and CM alone in the control group. The detailed study characteristics are presented in Table 1.

**Table 1:** Review characteristics.

**Author, year (Country)**	**Trials (subjects)**	**Treatment intervention**	**Control intervention**	**Quality assessment**	**Main results**
Taylor-Piliae and Finley (19)(American)	6 (229)	TC + CM	CM	Downs and Black Quality Index checklist	Among adults with CHF, TC was effective in improving exercise capacity and QoL, with less depression and B-type natriuretic peptide levels observed, when compared with controls. TC is a safe form of exercise and can be easily integrated into existing cardiac rehabilitation programs. Further research is needed with rigorous study designs and larger samples before widespread recommendations can be made.
Li et al. (20)(China)	7 (4,46)	TC + CM	CM	Cochrane criteria	TC can significantly improve the heart function and quality of life for the patients with heart failure, and this treatment could be applied to the rehabilitation process of patients with stable heart failure.
Wei et al. (21)(China)	10 (689)	TC + CM	CM	Cochrane criteria	The current evidence shows that TC is feasible for patients with heart failure as it has positive effects on life quality, physiological functions. Due to the limited quality and quantity of included studies, the above conclusion should be validated by more high quality studies.
Ren et al. (22)(China)	11 (656)	TC + CM	CM	Cochrane criteria	TC could improve 6MWD, quality of life and LVEF in patients with HF and may reduce BNP and HR. However, there is a lack of evidence to support TC altering other important long-term clinical outcomes so far. Further larger and more sustainable RCTs are urgently needed to investigate the effects of TC.
Gu et al. (23)(China)	10 (904)	TC + CM	CM	Cochrane criteria	Despite heterogeneity and risk of bias, this meta-analysis further confirms that TC may be an effective cardiac rehabilitation method for patients with chronic heart failure. Larger, well-designed RCTs are needed to exclude the risk of bias.
Pan et al. (24)(China)	4 (242)	TC + CM	CM	Jadad	TC may improve quality of life in patients with CHF and could be considered for inclusion in cardiac rehabilitation programs. However, there is currently a lack of evidence to support TC altering other important clinical outcomes. Further larger RCTs are urgently needed to investigate the effects of TC.

### Results on Review Quality Assessment

#### Methodological Quality

The results of AMSTAR-2 assessment are presented in Table 2. Since all SRs/MAs had more than one critical weakness (items 2, 4, 7, 9, 11, 13, and 15), their qualities were rated critically low. The key factors affecting the quality of the SRs/MAs on the AMSTAR-2 were the following: none of the SRs explicitly stated that the review methods were established before the conduct of the review and justified significant deviations from the protocol; none of the SRs provided a list of excluded studies and justified the exclusions.

**Table 2:** Result of the AMSTAR-2 assessments.

**Author, year**	**AMSTAR-2**																**Quality**
	**Q1**	**Q2**	**Q3**	**Q4**	**Q5**	**Q6**	**Q7**	**Q8**	**Q9**	**Q10**	**Q11**	**Q12**	**Q13**	**Q14**	**Q15**	**Q16**	
Taylor-Piliae and Finley (19)	Y	PY	Y	Y	Y	Y	N	Y	Y	N	Y	Y	Y	Y	Y	N	CL
Li et al. (20)	Y	PY	Y	PY	Y	Y	N	Y	Y	N	Y	Y	Y	Y	Y	N	CL
Wei et al. (21)	Y	PY	Y	Y	Y	Y	N	Y	Y	N	Y	Y	Y	Y	N	N	CL
Ren et al. (22)	Y	PY	Y	Y	Y	Y	N	Y	Y	Y	Y	Y	Y	Y	Y	Y	CL
Gu et al. (23)	Y	PY	Y	Y	Y	Y	N	Y	Y	Y	Y	Y	Y	Y	Y	Y	CL
Pan et al. (24)	Y	PY	Y	Y	Y	Y	N	Y	Y	N	Y	Y	Y	Y	Y	N	CL

*Y, Yes; PY, partial Yes; N, No; CL, Critically low; L, Low; H, High*.

#### Report Quality

The results of PRISMA checklist assessment are presented in Table 3. The results showed that the reporting was relatively complete, the section of title, abstract, introduction, and discussion were all well-reported (100%), but there were still some reporting flaws in other section. In section of methods, Q5 (topic of protocol and registration), and Q15 (risk of bias across studies) were reported inadequately (<50%); in section of results, Q22 (risk of bias across studies), Q23 (additional analyses) were reported inadequately (66.7%); in section of funding, Q27 (funding) was reported inadequately (33.3%). More details are summarized in Table 3.

**Table 3:** Result of the PRISMA assessments.

**Section/Topic**	**Items**	**Taylor-Piliae and Finley (19)**	**Li et al. (20)**	**Wei et al. (21)**	**Ren et al. (22)**	**Gu et al. (23)**	**Pan et sl. (24)**	**Compliance (%)**
Title	Q1. Title	Y	Y	Y	Y	Y	Y	100%
Abstract	Q2. Structured summary	Y	Y	Y	Y	Y	Y	100%
Introduction	Q3. Rationale	Y	Y	Y	Y	Y	Y	100%
	Q4. Objectives	Y	Y	Y	Y	Y	Y	100%
Methods	Q5. Protocol and registration	N	N	N	N	N	N	0%
	Q6. Eligibility criteria	Y	Y	Y	Y	Y	Y	100%
	Q7. Information sources	Y	Y	Y	Y	Y	Y	100%
	Q8. Search	Y	PY	Y	Y	Y	Y	83.3%
	Q9. Study selection	Y	Y	Y	Y	Y	Y	100%
	Q10. Data collection process	Y	Y	Y	Y	Y	Y	100%
	Q11. Data items	Y	Y	Y	Y	Y	Y	100%
	Q12. Risk of bias in individual studies	Y	Y	Y	Y	Y	Y	100%
	Q13. Summary measures	Y	Y	Y	Y	Y	Y	100%
	Q14. Synthesis of results	Y	Y	Y	Y	Y	Y	100%
	Q15. Risk of bias across studies	N	Y	N	Y	Y	Y	33.3%
	Q16. Additional analyses	N	Y	Y	Y	Y	Y	83.3%
Results	Q17. Study selection	Y	Y	Y	Y	Y	Y	100%
	Q18. Study characteristics	Y	Y	Y	Y	Y	Y	100%
	Q19. Risk of bias within studies	Y	Y	Y	Y	Y	Y	100%
	Q20. Results of individual studies	Y	Y	Y	Y	Y	Y	100%
	Q21. Synthesis of results	Y	Y	Y	Y	Y	Y	100%
	Q22. Risk of bias across studies	N	Y	N	Y	Y	Y	66.7%
	Q23. Additional analysis	N	Y	Y	Y	Y	Y	66.7%
Discussion	Q24. Summary of evidence	Y	Y	Y	Y	Y	Y	100%
	Q25. Limitations	Y	Y	Y	Y	Y	Y	100%
	Q26. Conclusions	Y	Y	Y	Y	Y	Y	100%
Funding	Q27. Funding	N	N	N	Y	Y	N	33.3%

#### Risk of Bias

For ROBIS, all SRs/MAs were at low risk in Phase 1 (assessing relevance), Domain 1 (study eligibility criteria) and Domain 3 (collection and study appraisal). All SRs/MAs were at high risk in Domain 2 (study eligibility criteria). Five SRs/MAs were rated low risk in Domain 4 (synthesis and findings), and 6 low risk in Phase 3 (risk of bias in the review). More details are presented in Table 4.

**Table 4:** Result of the ROBIS assessments.

**Reviews**	**Phase 1**	**Phase 2**				**Phase 3**
	**Assessing relevance**	**Domain 1: study eligibility criteria**	**Domain 2: identification and selection of studies**	**Domain 3: collection and study appraisal**	**Domain 4: synthesis and findings**	**Risk of bias in the review**
Taylor-Piliae and Finley (19)	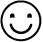	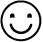	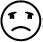	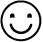	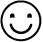	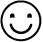
Li et al. (20)	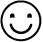	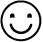	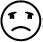	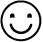	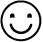	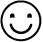
Wei et al. (21)	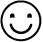	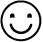	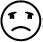	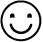	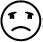	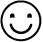
Ren et al. (22)	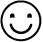	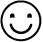	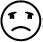	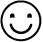	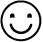	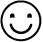
Gu et al. (23)	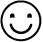	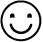	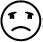	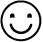	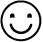	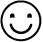
Pan et al. (24)	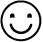	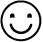	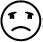	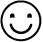	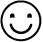	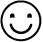

*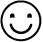, low risk; 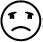, high risk*.

#### Evidence Quality

The results of GRADE assessment are presented in Table 5. The 6 SRs/MAs included 29 outcomes related to the effectiveness of TC for CHF. Among these outcome indicators, the quality of evidence was high in 1, moderate in 4, low in 15 and very low in 9. Risk of bias (*n* = 19) was the most common of the downgrading factors, followed by inconsistency (*n* = 17), imprecision (*n* = 16), publication bias (*n* = 9) and indirectness (*n* = 0).

**Table 5:** Results of evidence quality.

**Review**	**Outcomes**	**Certainty assessment**							**No. of patients**		**Relative effect (95% CI)**	***P*-value**	**Quality**
		**No. of trails**	**Design**	**Limitations**	**Inconsistency**	**Indirectness**	**Imprecision**	**Publication bias**	**Experimental**	**Control**			
Taylor-Piliae and Finley (19)	6-MWT	5	Rct	No	No	No	Serious^c^	No	135	134	SMD 0.353 (0.041, 0.664)	0.026	⊕⊕⊕⊕○ Moderate
	QoL	5	Rct	No	No	No	Serious^c^	No	135	134	SMD −0.671 (−0.864, −0.370)	0.000	⊕⊕⊕⊕○ Moderate
	BNP	4	Rct	No	No	No	Serious^c^	No	103	103	SMD −0.333 (−0.604, −0.062)	0.016	⊕⊕⊕⊕○ Moderate
Li et al. (20)	LVEF	3	Rct	No	Serious^b^	No	Serious^c^	No	128	108	MD 8.38 (6.98, 9.78)	<0.0001	⊕⊕⊕○○ Low
	6-MWT	5	Rct	No	No	No	No	No	161	151	SMD 0.85 (0.61, 1.08)	<0.0001	⊕⊕⊕⊕⊕ High
	QoL	4	Rct	No	Serious^b^	No	Serious^c^	No	131	122	SMD −1.10 (−1.91, −0.29)	0.008	⊕⊕⊕○○ Low
	NT-proBNP	2	Rct	No	No	No	Serious^c^	Serious^d^	45	45	SMD −12.14 (−23.78, −0.50)	0.04	⊕⊕⊕○○ Low
Wei et al. (21)	QoL	7	Rct	Serious^a^	Serious^b^	No	No	No	279	270	MD −9.37 (−13.09, −5.65)	<0.0001	⊕⊕⊕○○ Low
	6-MWT	7	Rct	Serious^a^	Serious^b^	No	No	No	277	267	MD 40.37 (9.48, 71.27)	0.01	⊕⊕⊕○○ Low
	LVEF	5	Rct	Serious^a^	Serious^b^	No	No	No	212	202	MD 7.89 (3.01, 12.77)	0.002	⊕⊕⊕○○ Low
	BNP	5	Rct	Serious^a^	No	No	No	No	162	162	MD −10.75 (−13.20, −8.30)	<0.0001	⊕⊕⊕⊕○ Moderate
	Peak VO_2_	3	Rct	Serious^a^	No	No	Serious^c^	Serious^d^	73	73	MD 0.29 (−1.23, 1.81)	0.71	⊕⊕○○○ Very low
	SBP	4	Rct	Serious^a^	No	No	Serious^c^	Serious^d^	80	81	MD −2.81 (−8.52, 2.90)	0.33	⊕⊕○○○ Very low
	DBP	3	Rct	Serious^a^	No	No	Serious^c^	Serious^d^	70	71	MD 0.37 (−3.73, 4.48)	0.86	⊕⊕○○○ Very low
Ren et al. (22)	6-MWT	7	Rct	Serious^a^	Serious^b^	No	No	No	241	233	WMD 65.29 (−32.55, 98.04)	<0.001	⊕⊕⊕○○ Low
	QoL	7	Rct	Serious^a^	Serious^b^	No	No	No	236	230	WMD −11.52 (−16.5, −6.98)	<0.001	⊕⊕⊕○○ Low
	BNP	5	Rct	Serious^a^	Serious^b^	No	No	No	133	133	SMD −1.08 (−1.91, −0.26)	<0.001	⊕⊕⊕○○ Low
	LVEF	5	Rct	Serious^a^	Serious^b^	No	No	No	200	180	WMD 9.94% (6.95, 12.93)	<0.001	⊕⊕⊕○○ Low
	HR	2	Rct	Serious^a^	No	No	Serious^c^	Serious^d^	38	38	WMD −2.52 (−3.49, −1.55)	<0.001	⊕⊕○○○ Very low
Gu et al. (23)	6-MWT	10	Rct	Serious^a^	Serious^b^	No	No	No	344	379	WMD 51.01 (30.49, 71.53)	<0.001	⊕⊕⊕○○ Low
	QoL	8	Rct	Serious^a^	Serious^b^	No	No	No	280	318	WMD −10.37 (−14.43, −6.32)	<0.001	⊕⊕⊕○○ Low
	LVEF	7	Rct	Serious^a^	Serious^b^	No	No	No	283	306	WMD 7.72% (3.58, 11.89)	0.003	⊕⊕⊕○○ Low
	BNP	6	Rct	Serious^a^	Serious^b^	No	No	No	178	221	SMD −1.01(−1.82, −0.19)	0.02	⊕⊕⊕○○ Low
Pan et al. (24)	6-MWT	3	Rct	Serious^a^	No	No	Serious^c^	No	95	95	MD 46.73 (−1.62, 95.09)	0.06	⊕⊕⊕○○ Low
	QoL	3	Rct	No	Serious^b^	No	Serious^c^	No	90	92	WMD −14.54 (−23.45, −5.63)	0.001	⊕○○○○ Very low
	BNP	2	Rct	No	Serious^b^	No	Serious^c^	Serious^d^	45	45	MD −61.16 (−179.27, −56.95)	0.31	⊕⊕○○○ Very low
	SBP	2	Rct	Serious^a^	Serious^b^	No	Serious^c^	Serious^d^	55	57	MD −1.06 (−13.76, 11.63)	0.87	⊕○○○○ Very low
	DBP	2	Rct	Serious^a^	Serious^b^	No	Serious^c^	Serious^d^	55	57	MD −0.08 (−3.88, 3.73)	0.97	⊕○○○○ Very low
	Peak VO_2_	2	Rct	No	No	No	Serious^c^	Serious^d^	65	65	MD 0.19 (−0.74, 1.13)	0.68	⊕○○○○ Very low

*CI, Confidence interval; WMD, weighted mean difference; MD, mean difference; SMD, standardized mean difference*.

a *The experimental design had a large bias in random, distributive findings or was blind*.

b *The confidence interval overlap less, the heterogeneity test P was very small, and the I^2^was larger*.

c *The Confidence interval was not narrow enough, or the simple size is too small*.

d *Funnel graph asymmetry, or fewer studies were included and there may have been greater publication bias*.

#### Outcomes and Efficacy Evaluation

A narrative synthesis was conducted for exercise capacity, QoL, BNP, NT pro-BNP, LVEF, peak VO_2_, SBP, DBP, and HR, as at least 2 studies assessed these outcomes. When TC was compared with controls, a significant effect for better QoL in all reviews (19–24), a significant effect for better exercise capacity in 5 reviews (19–23), a significant effect for lower BNP or NT pro-BNP in 5 reviews (19–23), a significant effect for better LVEF in 4 reviews (20–23), a significant effect for better HR in 1 review (22). However, no significant difference in peak VO_2_, SBP, and DBP between the TC and controls in 2 reviews (21, 24). More details are presented in Table 5.

## Discussion

A systematic overview of SR/MA is a comprehensive research approach for reassessing a comprehensive collection of SRs/MAs related to the same disease or health problem (25). An overview enables a more comprehensive integration of evidence, thus providing clinicians with higher quality evidence (25). Although there are an increasing number of publications of SR/MA on TC for CHF, the quality of those publications taken together has not been assessed until now. Therefore, an overview of this issue is needed. A literature search revealed that no overview of TC for CHF has been published to date.

### Summary of Main Findings

As a form of low-intensity physical activity originating in China, TC has gained popularity in Western countries as an alternative form of exercise in recent decades. Publications of SRs/MAs on TC for CHF is increasing annually. The included SRs/MAs on TC for CHF in this current overview were published from 2013 to 2020, and 83.3% of them were published after 2017, possibly indicating that TC has begun to attract attention as an alternative form of exercise for CHF. This overview included 6 SRs/MAs, all of which reached positive conclusions of TC for CHF; however, the authors did not want to draw firm conclusions due to the small size of the included RCTs or their low quality. Moreover, according to the evaluation results of AMSTAR-2, PRISMA, ROBIS, and GRADE, the quality of the SRs/MAs and the evidence quality of the outcome measures are generally unsatisfactory, indicating that the results of included SRs/MAs may be very different from the real situation. Therefore, based on the above findings of past SRs/MAs, we cannot draw a firm conclusion on TC for CHF, but results suggest that TC is a promising complementary treatment for CHF.

### Implications for Practice and Research

Dyspnea and fatigue limit exercise capacity in CHF patients, leading to progressive deconditioning and exercise intolerance, resulting in a vicious cycle of worsening dyspnea and fatigue (24). Furthermore, various physical and emotional symptoms that CHF patients experience could limit their physical and social activities and result in poor QoL. Therefore, Cardiac rehabilitation (with exercise training at its core) is highly desirable for patients with CHF (8). TC is a promising adjunct to exercise-based cardiac rehabilitation for adults with CHF (10). As a mind-body integrated exercise, TC including mind peace, breath flow, body movement, could activate the natural self-healing ability, evoke the balanced release of endogenous neurohormones and various natural health recovery mechanisms, thereby improving cardiac collateral circulation and increasing activity tolerance (26). Moreover, as a moderate intensity exercise, TC could improve the degree of parasympathetic nerve, inhibit sympathetic nerve activity, increase the coronary collateral circulation, cardiac stroke volume, and cardiac output, thereby achieving increased LVEF (22). The mechanism of TC practice may be to maintain the balance of “Yin” and “Yang,” which was a contradiction of unity. When CHF patients perform TC, they should pay attention to the regulation of body shape, spirit and significance, and qi, so that the body enters a relaxed state; this could be achieved by adjusting the balance of autonomic nerves and reduce the sympathetic nervous tension, thereby adjusting breathing, slowing HR and improving the strength and body reactivity (22). Therefore, TC may inhibit adrenergic nervous system, decrease sympathetic nervous system, and slow HR to improve CHF.

Assessment of various aspects of the included SRs/MAs using the AMSTAR-2, PRISMA, and ROBIS identified areas for common improvement. For example, they all ignored the need to register the protocol, provided a list of excluded studies. Though the quality was unsatisfactory, meanwhile it also means that there was much room to address the quality during the SRs/MAs process. For evidence quality with GRADE, we found that risk of bias within the original RCTs was the most common of the downgrading factors in the included SRs/MAs, all of the outcome indicators were demoted because of the limitations caused by bias in random, distributive hiding or blind. Therefore, the assessment results may help guide future high-quality studies.

### Strength and Limitations

To the best of our knowledge, this current study is the first systematic overview to explore the evidence of TC for CHF. Based on the current results, the quality of the SRs/MAs and evidence quality of outcome indicators are presented cleanly, which may have certain reference value for the clinical practice and research of TC in the treatment of CHF. However, due to the generally low quality of SRs/MAs and outcome indicators, firm conclusions were impossible to draw, caution is warranted when recommending Tai Chi as a complementary treatment for CHF.

## Conclusion

TC may be a promising complementary treatment for CHF. However, the quality of past SRs/MAs is limited, further rigorous, comprehensive SRs/MAs and RCTs that adhering to the guidelines are required to provide robust evidence for definitive conclusions.

## Data Availability Statement

The original contributions presented in the study are included in the article/supplementary material, further inquiries can be directed to the corresponding author/s.

## Author Contributions

JH planned and designed the study, and drafted the manuscript. MS and XQ screened potential studies and extracted data from the included studies. MS, XQ, and YX assessed the reviews. YH provided guidance on the overview methodology. All authors read, critically reviewed, and approved the final manuscript as submitted.

## Conflict of Interest

The authors declare that the research was conducted in the absence of any commercial or financial relationships that could be construed as a potential conflict of interest.

## Abbreviations

TC: Tai chi
CHF: chronic heart failure
SR: systematic review
MA: Meta-analysis
AMSTAR-2: Assessing the Methodological Quality of Systematic Reviews 2
PRISMA: Preferred Reporting Items for Systematic Reviews and Meta-Analyses
ROBIS: Risk of Bias in Systematic reviews
GRADE: Grading of Recommendations, Assessment, Development, and Evaluation
RCT: random control trails
CM: conventional medication
6MWD: 6-min walk distance
Qol: quality of life
MLHF: Minnesota Living with Heart Failure Questionnaire
BNP: B-type natriuretic peptide
NT pro-BNP: N-terminal pro brain natriuretic peptide
LVEF: left ventricular ejection fraction
peak VO_2_: peak oxygen uptake
SBP: systolic blood pressure
DBP: diastolic blood pressure
HR: heart rate.
